# Dicarbonyls and Advanced Glycation End-Products in the Development of Diabetic Complications and Targets for Intervention

**DOI:** 10.3390/ijms18050984

**Published:** 2017-05-05

**Authors:** Sebastian Brings, Thomas Fleming, Marc Freichel, Martina U. Muckenthaler, Stephan Herzig, Peter P. Nawroth

**Affiliations:** 1 Department of Medicine I and Clinical Chemistry, University Hospital Heidelberg, 69120 Heidelberg, Germany; thomas.fleming@med.uni-heidelberg.de (T.F.); peter.nawroth@med.uni-heidelberg.de (P.P.N.); 2 Department of Nuclear Medicine, University Hospital Heidelberg, 69120 Heidelberg, Germany; 3 German Center for Diabetes Research (DZD), 85764 Munich-Neuherberg, Germany; 4 Joint Heidelberg-IDC Translational Diabetes Program, Helmholtz Center, 85764 Munich-Neuherberg, Germany; stephan.herzig@helmholtz-muenchen.de; 5 Institute of Pharmacology, University of Heidelberg, 69120 Heidelberg, Germany; marc.freichel@pharma.uni-heidelberg.de; 6 Molecular Medicine Partnership Unit, University of Heidelberg, 69120 Heidelberg, Germany; martina.muckenthaler@med.uni-heidelberg.de; 7 Institute for Diabetes and Cancer (IDC), Helmholtz Center, 85764 Munich-Neuherberg, Germany

**Keywords:** advanced glycation end-products, diabetes, glyoxalase, aldose reductase, methylglyoxal, glyoxal, 3-deoxyglucosone

## Abstract

Advanced glycation end-products (AGEs) are non-enzymatic protein and amino acid adducts as well as DNA adducts which form from dicarbonyls and glucose. AGE formation is enhanced in diabetes and is associated with the development of diabetic complications. In the current review, we discuss mechanisms that lead to enhanced AGE levels in the context of diabetes and diabetic complications. The methylglyoxal-detoxifying glyoxalase system as well as alternative pathways of AGE detoxification are summarized. Therapeutic approaches to interfere with different pathways of AGE formation are presented.

## 1. Introduction

Diabetes is characterized by elevated blood glucose levels and over 400 million people are suffering from diabetes worldwide [1]. Despite adjustment of blood glucose levels, diabetic complications develop frequently. These are a major cause of death, with estimated 4 million diabetes related deaths per year worldwide [1,2]. Glycation, the non-enzymatic post-translational modification of proteins is enhanced in diabetes and is associated with the development of diabetic complications [3,4]. In addition to proteins, the modification of DNA has also been described [5,6]. Advanced glycation end-products (AGEs) are the resulting modifications and form via a number of pathways [7,8]. The main precursors of AGEs are glucose and reactive dicarbonyls. Originally, AGE formation was discovered in the context of food preparation and these exogenous AGEs can contribute to the development of diabetes in addition to AGEs which originate in vivo [9,10,11]. The focus of the current review is on the in vivo formation and detoxification pathways of AGEs and their precursors in the context of diabetes. The importance of dicarbonyls and dicarbonyl-derived AGEs for the development of diabetic complications as well as potential therapeutic interventions are discussed. 

## 2. Glucose and Dicarbonyl Dependent Advanced Glycation End-Product Formation

**Figure 1:**
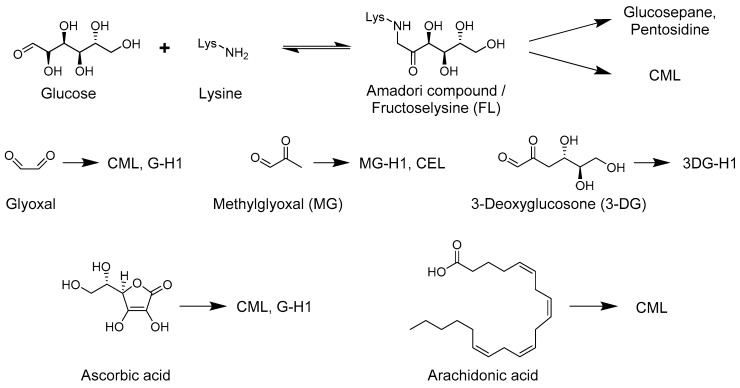
Shown are the major precursors for the advanced glycation end-products (AGEs) identified in vivo. 3DG-H1: 3-Deoxyglucosone-hydroimidazolone 1; CML: *N*^ε^-(carboxymethyl)-lysine; G-H1; Glyoxal-derived hydroimidazolone 1; MG-H1: Methyglyoxal-derived hydroimidazolone 1.

The main pathways of AGE formation involve the reaction of glucose or dicarbonyls with primary amines (*N*-terminal or lysine side chain) or the guanidine group of the arginine side chain (Figure 1). Other AGE precursors include polyunsaturated fatty acids as well as ascorbic acid [12,13].

**Figure 2:**
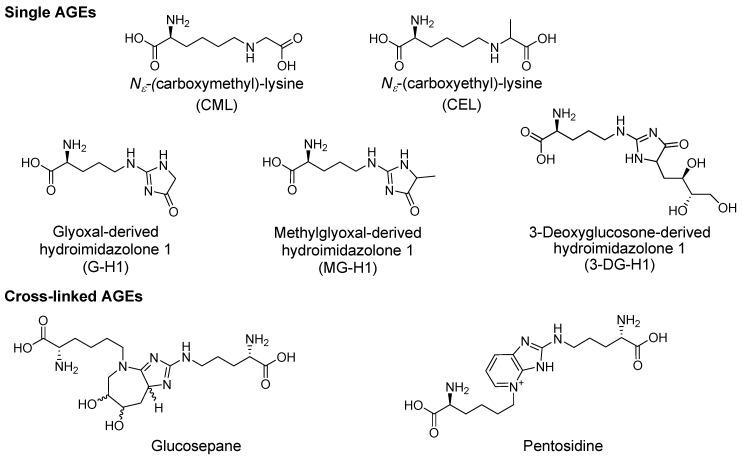
The main AGEs that have been quantified in vivo are shown. AGE structures are given as AGE free adducts.

According to the classical view of the Maillard reaction [11], glucose reacts with a primary amine which is followed by a series of rearrangements and/or fragmentation reactions [14,15,16] to yield the final AGEs; a process which takes days to weeks in a physiological setting [17,18]. *N*^ε^_-_(carboxymethyl)-lysine (CML; see AGEs in Figure 2) was first detected in human urine in 1975 [19]. It was subsequently described as the first AGE by proving formation from the glucose-lysine adduct fructoselysine in 1986 [14]. Additional pathways which yield CML include the autoxidation of aldoses and ketoses [20], and the precursors ascorbic acid [12], polyunsaturated fatty acids [13] as well as the dicarbonyl pathway discussed in the following section. The main cross-linking AGE glucosepane forms via a glucose-lysine adduct which subsequently reacts with the guanidine side chain of arginine under physiological conditions [21,22]. 

The other major glycation pathway, which is now also classified as part of the Maillard reaction [7], proceeds via the dicarbonyls methylglyoxal (MG), glyoxal and 3-deoxyclucosone (3-DG), of which MG is the most reactive compound [23]. Dicarbonyls form via several pathways some of which are intrinsically linked with the glucose derived pathway of AGE formation. Indeed, the glycolysis intermediates glyceraldehyde-3-phosphate and dihydroxyacetonephosphate are important precursors for MG [24]. Glucose-independent precursors of MG derive from the fatty acid and amino acid metabolism as well as from ascorbic acid [25,26]. Glyoxal formation proceeds via transition metal catalyzed fragmentation of glucose [27] and glucose amine adducts including fructoselysine (FL) [15,16,28] as well as peroxidation of polyunsaturated fatty acids [29]. 3-DG forms from glucose adducts to *N*-terminal amines or lysine side chains termed Amadori compound [30] as well as from fructose-3-phosphate [31]. Interestingly the latter compound forms after phosphorylation of the Amadori compound by fructosamine-3-kinase, in an enzymatic reaction termed deglycation [32]. 

The peroxidation of fatty acids can lead to the production of numerous dicarbonyls including malondialdehyde (MDA) and the aldehyde 4-hydroxynonenal (HNE) [8]. Relatively few studies have investigated changes of peroxidation specific precursors and subsequent amino acid modifications, termed advanced lipoxidation end-products (ALEs) in diabetes [33,34]. Furthermore, little is known about the relative abundance of ALEs compared to AGEs since only few comparative studies have been carried out. In one study, no MDA and HNE lysine adduct could be quantified by gas chromatography combined with mass spectrometry (GC–MS) while CML was quantified easily [35]. One issue with lipid peroxidation specific dicarbonyls and ALEs is their detection; mass spectrometry based methods as described in more detail in Section 5 for glucose dependent dicarbonyls and AGEs have been reported but they are labor intensive, involving immunopurification and other enrichment steps, which makes quantification difficult [36,37]. 

Dicarbonyls are more reactive than glucose making them relevant glycating agents despite the overall low dicarbonyl concentration found in tissue. The methylglyoxal-derived AGE, methylglyoxal-derived hydroimidazolone 1 (MG-H1) rapidly forms at the guanidine group of arginine [38]. In line with these findings, MG-H1 was reported as the most abundant AGE in numerous studies alongside the glucose-derived lysine arginine cross-link glucosepane (6-[2-{[(4*S*)-4-ammonio-5-oxido-5-oxopentyl]amino}-6,7-dihydroxy-6,7,8,8a-tetrahydroimidazo-[4,5-*b*]-azepin-4(5*H*)-yl]-l-norleucinate) and CML [3,18,39,40]. Levels of the 3-DG derived arginine adduct 3-deoxyglucosone-derived-hydroimidazolone 1 (3-DG-H1) are also comparatively high but the compound has been quantified in fewer studies [39]. Additional abundant dicarbonyl derived AGEs are the MG-lysine adduct *N*^ε^-(carboxyethyl)lysine (CEL) and glyoxal-derived hydroimidazolone (G-H1 also abbreviated Glarg) [3,39,41]. In addition to protein-AGE adducts, modification of DNA bases by MG has been reported [42]. The resulting products of deoxyguanosine modification are *N*2-(1-carboxyethyl)-2′deoxyguanosine (CEdG) and two structural isomers 3-(2′-deoxyribosyl)6,7-dihydro-6,7-dihydroxy-6/7-methylimidazo-[2,3-*b*]purine-9(8)one (MGdG) while deoxyadenosine modification results in *N*2-(1-carboxyethyl)-2′-deoxyadenosine (CEdA) [5,43]. Glyoxal modification of deoxyguanosine results in the adduct 3-(2′-deoxyribosyl)-6,7-dihydro-6,7-dihydroxyimidazo-[2,3-*b*]purine-9(8)one (GdG) [5]. 

It is worth noting that, while formation of the major cross-link glucosepane starts with a glucose-lysine adduct, the pathway features an intermediate dicarbonyl structure [44]. The same is the case for pentosidine ((2*S*)-2-amino-6-[2-[[(4*S*)-4-amino-4-carboxybutyl]amino]imidazo[4,5-*b*]-pyridine-4-yl]hexanoic acid), a less abundant lysine–arginine cross-link [22]. Consequently, a large proportion of AGE intermediates, including the most abundant cross-link feature a dicarbonyl structure. Thus, glucose is an important precursor while the subsequent AGE formation frequently proceeds via free or protein bound dicarbonyl intermediates.

## 3. Interpretation of Advanced Glycation End-Product Levels In Vivo

The mechanism of AGE formation has been investigated in numerous in vitro studies and many different pathways have been discovered. For example CML can form via metal-catalyzed oxidative cleavage of the Amadori compound [14], polyunsaturated fatty acids [13] or ascorbic acid [12] as well as from glyoxal [15,28] (Figure 1). The mechanism of AGE formation has been summarized in an extensive review recently [7]. In diabetes, elevated levels of AGEs have mostly been attributed to enhanced precursor levels such as glucose and dicarbonyls. In this section, we briefly summarize factors that affect AGE levels in vivo.

The amount of AGE protein adducts in vivo depends on the half-life of the protein, the rate of AGE formation and the chemical stability of the resulting AGE [45,46,47]. While AGEs overall are rather stable, there are differences: Both CML and CEL are recovered even after acid hydrolysis of proteins [14,48]. Glucosepane is stable for at least three weeks under physiologic conditions [22]. The half-life of MG-H1 was reported to be around 2–3 weeks under physiologic conditions [49,50].

The rate of AGE formation on each specific protein depends on the level of the precursor and the number of potential sites for modification. Only certain arginine and lysine residues of proteins are modified, supporting the notion that the primary and/or the secondary structure affect site reactivity. For example human serum albumin (HSA) contains 24 arginine residues, of which only five are modified with MG-H1 upon incubation with MG in vitro while one of these sites was particularly reactive [51]. A similar specificity is seen for the glycation of lysine residues of HSA [52]. However, considering the overall low amount of modified residues and the large number of reactive sites this is unlikely to be a limiting factor in vivo [39]. In addition to the precursor level, the formation of certain AGEs such as CML is catalyzed by transition metals which consequently can affect the rate of formation [15,53,54].

CML was first identified on long lived proteins and a long protein half-life was deemed necessary for AGE-protein adducts in part due to the slow formation of AGEs via the Maillard reaction [20,45,55]. The great difference in reactivity at certain sites due to primary and secondary structure as well as the fast AGE formation pathway via reactive dicarbonyls explains why short-lived proteins are modified as well. It follows that a large amount of AGE-modified proteins are removed by proteolysis during normal turnover resulting in the release of non-protein bound AGEs, termed AGE free adducts. 

**Figure 3:**
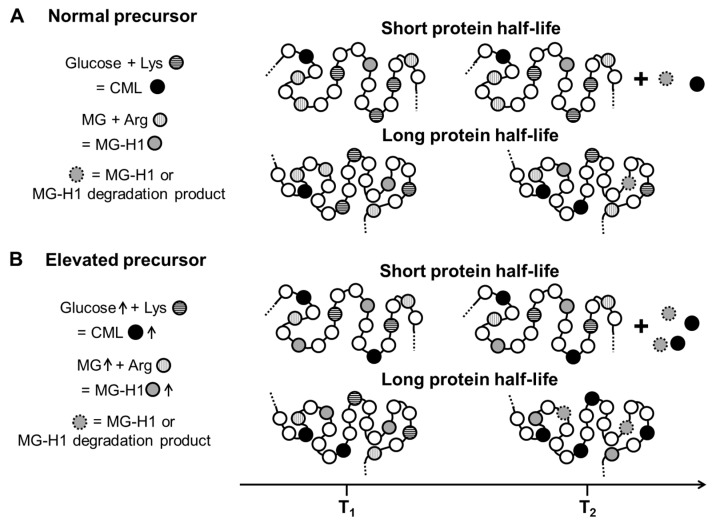
Influence of protein half-life, chemical stability of AGEs and precursor level on the accumulation of AGE protein adducts over time. AGE formation under normal precursor conditions are shown in (**A**). Proteins with short half-life do not show long-term protein–AGE accumulation but AGE free adducts are released upon proteolysis. Stable AGEs such as CML accumulate over time on proteins with long half-life. The effect of an elevation of precursor levels as it is found in diabetes is shown in (**B**).

An overview of the kinetics of AGE formation on proteins with a short and a long half-life is shown in Figure 3. Differences between normal and high precursor conditions, frequently encountered in diabetes are shown exemplary for one stable AGE (CML) and one labile AGE (MG-H1). Accumulation of stable AGEs over time occurs on long-lived proteins while modification of short-lived proteins results mainly in elevation of AGE free adducts [47,56]. Furthermore, labile AGEs such as MG-H1 should not accumulate significantly over time. This is supported by data from skin collagen which has a very long half-life where CML levels but not MG-H1 is strongly associated with donor age [56]. However, this is not true for all tissues as a strong correlation with age is seen for MG-H1 levels in lenses from normal donors [7,40]. This could be explained by a significant increase in the rate of precursor formation, namely MG, due to elevated production or an impaired detoxification mechanism. The study by Duran-Jimenez is also very interesting as it is one of the few where time dependent changes of different AGEs namely CML, MG-H1 and G-H1 as well as FL were investigated in a diabetic rat model [18]. While all compounds were elevated in diabetes, no time dependent increase was noted between 3 and 24 weeks of diabetes, possibly due to protein turnover.

## 4. Dicarbonyls in Diabetes and Relation to Diabetic Complications

The reactive dicarbonyls MG [57,58,59,60,61,62], 3DG [58,59,62,63,64] and glyoxal [57,59,60,62] are elevated in serum of diabetic patients alongside blood glucose levels. Protein bound AGEs and AGE free adducts are increased in experimental models of diabetes [18,39,65], and in diabetic patients in parallel with their precursors [66,67]. While glucose and intermediates of glucose metabolism are important dicarbonyl precursors, plasma levels of MG and glucose or glycated hemoglobin (HbA1c) levels do not necessarily correlate [26,68,69]. Reasons for the discrepancy between MG and blood glucose levels may be that additional MG precursor derive from the fatty acid and amino acid metabolism [26]. In addition changes in the enzymatic detoxification systems can contribute to altered dicarbonyl levels [70]. Consequently reducing the blood glucose level is likely to not be sufficient to correct the metabolic derangement and associated complications in diabetes. This is not to deny an essential role of glucose in the development of diabetic complications. This is supported by the findings that, in addition to HbA1c, the glucose-lysine adduct FL as well as the glucose derived cross-link glucosepane are strong predictors of diabetic complications [3]. 

### 4.1. Association of Methylglyoxal with Diabetic Complications

The association between MG and MG-derived AGEs with diabetic complications has been investigated in numerous experimental and clinical studies. It was found that skin levels of AGEs are associated with the progression of diabetic nephropathy, diabetic neuropathy and diabetic retinopathy in type 1 diabetic humans [3]. Interestingly, in this study the correlation of MG-H1, with the development of diabetic neuropathy was very strong even when corrected for all other risk factors. This provides support for the finding that MG levels are particularly high in diabetic patients with enhanced pain sensitivity while MG was shown to be causative of hyperalgesia in mice [62]. In a separate study, MG-H1 plasma levels correlated with the heat pain detection threshold in the foot further confirming the importance of MG in diabetic neuropathy [71]. However, another recent study found no correlation between serum MG levels and peripheral neuropathy in type 2 diabetic patients [72]. As the authors commented this does not rule out a role for MG in the pathogenesis of diabetic neuropathy. Levels of the MG-derived AGE MG-H1 are extraordinarily high in the sciatic nerve so that MG plasma levels may not be a good predictor of MG levels in the nerve [73]. In addition, MG is notoriously difficult to quantify so that measurement of free and protein bound MG-H1 may be a better marker for MG exposure [74]. High MG levels are also associated with a faster rate of cognitive decline in humans as a possible mechanistic link between neurodegenerative disorders and diabetes [75]. 

MG has been implicated in the development of diabetic nephropathy in addition. A recent study in 1481 type 2 diabetic patients reported positive association of serum MG levels with albumin/creatinine ratio (ACR) at baseline while changes in the estimated glomerular filtration rate were inversely associated with MG during follow up [76]. Similarly levels of urinary and plasma MG levels correlated with basement membrane thickness in two cohorts of patients while MG levels in red blood cells were higher in progressors vs. non-progressors of diabetic nephropathy upon incubation with glucose [68]. This was confirmed by another study, which reported the correlation of plasma MG levels with serum creatinine and ACR in type 2 diabetic patients [77]. In addition plasma levels of the AGE MG-H1 and another MG derived AGE, CEL discriminated between fast and slow progressors with diabetic nephropathy in patients with type 1 diabetes [78]. The data is in contrast to the analysis of skin AGEs in type 1 diabetic patients mentioned previously [3]. While MG-H1 levels correlated strongly with neuropathy in the latter study, no correlation with nephropathy was seen. One major difference between the two studies is the analysis of skin tissue vs. plasma samples. Local exposure towards AGE precursors may well be different in those tissues. Furthermore, the most abundant proteins in plasma and skin are albumin and collagen respectively [79]. MG-H1 has a chemical half-life of 2–3 weeks [49,50] similar to albumin half-life of 19 days [80] as opposed to skin collagen which has a half-life of 15 years [47]. Thus, MG-H1 levels of albumin but not of collagen are affected by protein turnover. 

MG levels are also positively related to intima media thickening and the elevation of blood pressure in diabetes supporting a link between MG and macroangiopathy [81]. The MG derived AGE, CEL also correlated with cardiovascular disease and all-cause mortality alongside the glucose derived AGEs pentosidine and CML [82]. This correlation was present even after correction for renal dysfunction, low-grade inflammation, endothelial dysfunction and arterial stiffness. 

MG modified deoxyguanosine namely CEdG has also been detected in urine of healthy volunteers [6]. CEdG was subsequently shown to be elevated in urine but not in plasma of type 2 diabetic patients alongside another MG deoxyguanosine adduct, MGdG [5]. Levels of CEdG were also elevated in glomeruli of patients with diabetic kidney disease [83] and of diabetic rats [84]. The relevance of MG modified DNA bases in vivo is currently not known. Transformation of cells with CEdG modified vectors vs. unmodified vectors resulted in a significant reduction of protein activity [85]. However, as with AGE modified proteins, care needs to be taken when transferring results from in vitro experiments with strongly modified substrates to an in vivo setting [86,87].

### 4.2. Association of 3-Deoxyclucosone and Glyoxal with Diabetic Complications

The majority of studies regarding dicarbonyls and dicarbonyl derived AGE formation have focused on MG. The other two dicarbonyls which have been investigated in the context of diabetes and AGE formation are 3-DG and glyoxal. Levels of the 3-DG derived AGE, 3-DG-H1, are present at similar levels as MG-H1 in human plasma and are elevated in experimental diabetes in the renal glomeruli the retina and the sciatic nerve [39]. Plasma levels of 3-DG also correlate with glomerular basement membrane thickness [68]. Interestingly, the correlation remained significant even after adjustment for HbA1c suggesting that factors in addition to glucose levels are responsible for 3-DG formation [68]. This is despite the fact that 3-DG forms mainly from glucose namely via the Amadori compound. One explanation for the discrepancy may be the activity of fructosamine-3-kinase which destabilizes glucose-amine adducts with resulting release of 3-DG [88]. Kusunoki et al. reported elevated serum levels of 3-DG in diabetic patients with normoalbuminuria and further elevations in those patients with microalbuminuria and overt proteinuria [63]. 

While levels of glyoxal [57,59,62] are elevated in diabetes a relation to diabetic complications is currently uncertain. Compared to 3-DG and MG derived AGEs, the glyoxal-derived AGE G-H1 is present at rather low levels, except for plasma and elevations in diabetes are less pronounced [3,39,41]. In line with these findings no correlation of G-H1 skin levels with diabetic nephropathy, diabetic neuropathy and diabetic retinopathy were seen [3]. In contrast the glyoxal-derived DNA adduct GdG was strongly elevated in plasma of diabetic patients. It is thus possible that glyoxal is relevant for the glycation of DNA rather than protein.

## 5. Quantification of Dicarbonyls and Advanced Glycation End-Products

Liquid chromatography coupled tandem mass spectrometry (LC–MS/MS) employing internal, preferably isotopically labeled, standards is the method of choice for the quantification of dicarbonyls and several methods have been reported [59,74,89,90]. Alternatively, the use of GC–MS and high-performance liquid chromatography (HPLC) methods has been reported for the detection of MG [91,92]. Proper handling of samples and derivatization prior to analysis is essential due to the high reactivity of the compounds and the potential of MG formation from precursors glyceraldehyde-3-phosphate and dihydroxyacetonephosphate during sample processing [74]. The most commonly employed derivatizing agent is o-phenylenediamine but other reagents such as *O*-(2,3,4,5,6-pentafluorobenzyl)hydroxylamine hydrochloride or the fluorescent product 1,2-diamino-4,5-dimethoxybenzene have also been used successfully [74,92,93].

The large number of AGEs in combination with the differences in stability is a challenge for quantification. The method of choice for the measurement of AGE-protein adducts is LC–MS/MS analysis with stable isotopic dilution [94]. LC-MS/MS methods have also been published for the quantification of MG-DNA adducts [43,95,96]. For the absolute quantification of AGE protein adducts, proteins need to be hydrolyzed. Due to the instability of certain AGEs, enzymatic hydrolysis rather than acid hydrolysis is employed frequently and different digestion protocols have been developed [40,97]. Problems that can occur with the enzymatic approach are incomplete hydrolysis or bacterial contamination of the sample due to prolonged incubation at 37 °C. Bacterial contamination can be prevented by inclusion of antibiotics. Incomplete hydrolysis can be overcome by custom tailoring the digestion protocols for the specific protein of interest, for example the inclusion of collagenase for isolated collagen or collagen-rich tissue [94]. 

While mass spectrometry is considered the method of choice, it requires expensive equipment and the access to standards. Not all of the latter are commercially available and may require lengthy synthesis [98]. Alternatively AGEs can be detected by immunoassays. However, while numerous anti-AGE antibodies are commercially available, they are often not specific and can cross-react with other AGEs or the non-glycated protein. For example the “CML-specific” monoclonal antibody 6D12 reacts with CEL as well [99]. To exclude such cross-reactivity, blocking experiments should be carried out in order to determine specificity of the antibodies ideally using the AGE antigen in a sequence different from the one against which the antibody was raised [65].

The cross-linking AGE pentosidine was discovered and could be quantitated early due to chemical stability and the autofluorescent properties despite the low levels of this compound in tissues [46]. However, since most AGEs are not fluorescent this method is of limited use for AGE detection overall. A similar problem is encountered with the measurement of skin autofluorescence as an analytical tool for AGEs in patients. In support for skin autofluorescence it correlated with the AGEs pentosidine, CML, CEL and mean HbA1c and was a strong predictor of cardiac mortality in diabetic patients [100,101]. The lack of fluorescence of major AGEs such as CML, glucosepane and MG-H1 as well as the interference with other fluorescent compounds such as nicotinamide adenine dinucleotide, flavine adenine dinucleotide and porphyrins are limitations of this method [97].

## 6. Pathways of Dicarbonyl and Advanced Glycation End-Product Metabolism

### 6.1. The Glyoxalase System

**Figure 4:**
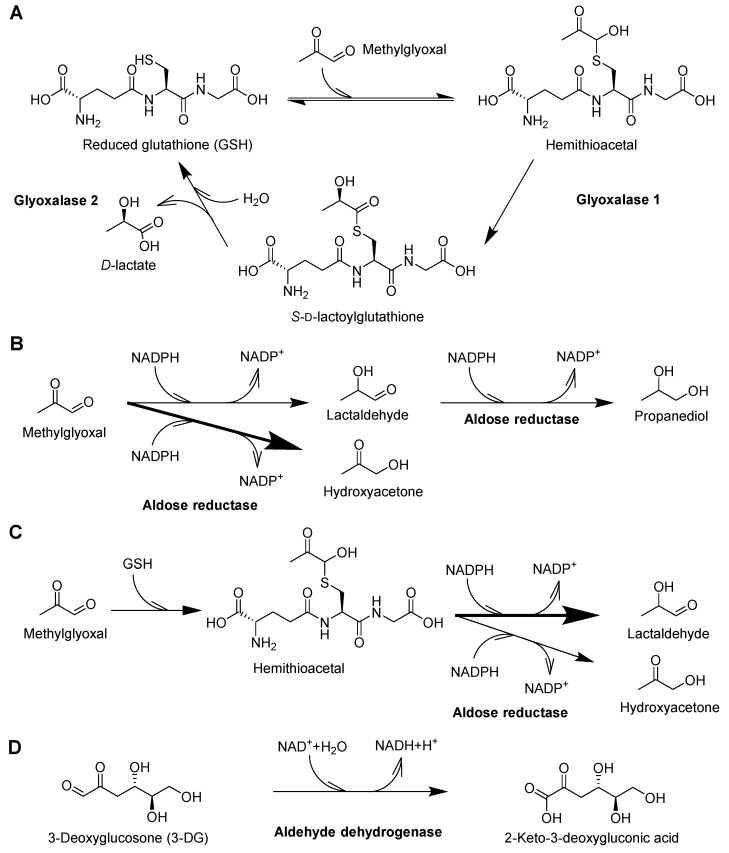
Pathways of dicarbonyl detoxification. MG reacts with glutathione to yield hemithioacetal, the substrate for glyoxalase 1 (GLO1). (**A**) The product, *S*-d-lactoylglutathione, is hydrolyzed by glyoxalase 2 (GLO2) to yield d-lactate and reduced glutathione. Glyoxal is also metabolized via this pathway and results in glycolate production (not shown). Aldose reductases (**B**) catalyze the NADPH (nicotinamide adenine dinucleotide phosphate)-dependent reduction of MG which yield hydroxyacetone (major product) and lactaldehyde (minor product) in the absence of reduced glutathione (GSH). Lactaldehyde may be further reduced to propanediol. In the presence of GSH (**C**) aldose reductase acts on the hemithioacetal which results in a shift towards lactaldehyde production. Products of 3-DG and glyoxal metabolism of aldose reductase are 3-deoxyfructose and glycolaldehyde respectively (not shown). Aldehyde dehydrogenase (**D**) oxidizes dicarbonyls and is of potential importance for 3-DG metabolism with the resulting product being 2-keto-3-deoxygluconic acid. Products of MG and glyoxal oxidation are pyruvate and glyoxylate, respectively (not shown).

The glyoxalase system is the best characterized pathway for the metabolism of MG (Figure 4A) and has been covered in a recent review [102]. According to some estimates it metabolizes over 99% of the produced MG [103]. Additional dicarbonyls which are metabolized via this pathway are glyoxal, phenylglyoxal and hydroxypyruvaldehyde but not 3-DG [104,105,106]. Two enzymes, glyoxalase 1 (GLO1) and glyoxalase 2 (GLO2), make up the glyoxalase system in eukaryotes and require a catalytic amount of glutathione. First, reduced glutathione (GSH) reacts with the aldehyde of the dicarbonyl resulting in a hemithioacetal followed by the GLO1 catalyzed formation of *S*-d-lactoylglutathione in the case of MG. The second reaction is catalyzed by GLO2 and results in the production of d-lactate and reduced glutathione. GLO1 is the rate-limiting enzyme and is ubiquitously expressed at high levels [107,108]. The detoxification of glyoxal proceeds accordingly, albeit at a slower rate, and results in the formation of glycolate [106].

Alterations in the glyoxalase system may contribute to the development of diabetic complications. Reduction of GLO1 by small interfering RNA is associated with elevated MG-H1 protein adducts and changes indicative of diabetic nephropathy namely an increased mesangial area, a thickened glomerular basement membrane as well as albuminuria [109]. In the same study, overexpression of GLO1 protected from diabetic nephropathy [109]. Similar results were obtained in streptozotocin (STZ)-induced diabetic rats where albuminuria was lower in GLO1 overexpressing animals in parallel to decreased MG levels and MG-H1 levels [70]. Literature supports higher [110] or unaltered GLO1 activity [111] in red blood cells of type 1 and type 2 diabetic patients while patients with complications are reported to have higher levels than those without [110,111]. The presence of higher d-lactate levels alongside elevated GLO1 activity lends further support to the findings that GLO1 activity is elevated in diabetes [110]. Such an increase in the GLO1 system could be an adaptive response to deal with elevated MG levels [112]. These findings suggest that a lowered capacity of the GLO1 system is not causative of elevated MG levels in diabetes but rather that the system is overwhelmed by the amount of MG produced. However elevated levels of GLO1 in lenses of diabetic mice were paralleled by high MG levels suggesting that GLO1 activity may not be sufficient in diabetes [113]. Most studies regarding the glyoxalase system have focused on MG and its metabolites and relatively little is known about other glyoxalase metabolites such as the glyoxal derived glycolate. In a study already published in 1975 it was shown that glycolate levels are increased in urine of fed as well as fasted STZ-diabetic rats [114]. Authors did not detect concomitant changes of other metabolites related to glycolate metabolism such as glyoxylate so that the elevation may be indicative of enhanced glyoxal metabolism via the glyoxalase system in these animals. 

### 6.2. Aldose Reductase

Aldose reductases are another group of dicarbonyl metabolizing enzymes (Figure 4B,C). Products of NADPH (nicotinamide adenine dinucleotide phosphate)-dependent MG reduction are hydroxyacetone and lactaldehyde or propanediol in the case of two subsequent reductions [115]. In the absence of GSH, hydroxyacetone is the major product while lactaldehyde is the major product when GSH is present [93,115]. In addition to MG, aldose reductase converts glyoxal to glycolaldehyde [116] and 3-DG to 3-deoxyfructose [117]. 

Due to the high activity of the glyoxalase system it has been questioned whether aldose reductase is relevant for the detoxification of dicarbonyls in vivo. The effect of GSH levels on aldose reductase activity (AKR1B1) and GLO1 activity was investigated in this context in humans [115]. It was concluded that AKR1B1 assists GLO1 in the detoxification of MG in tissue where it is highly expressed and GSH levels are low. In an early study aldose reductase overexpression protected from MG as well as 3-DG-induced toxicity [118]. Another study reported a compensatory increase of aldose reductase activity (Akr1b3) in murine Schwann cells when GLO1 was knocked out [93]. These cells displayed MG levels and MG-H1 levels similar to wild type but were more sensitive towards external stimulation with MG compared to wild type cells. Moreover, the effect of aldose reductase knockout (AKR1B3) has been investigated in hearts of diabetic mice as well [119]. Mice not expressing the enzyme had higher levels of MG and glyoxal derived AGEs. Based on simulation experiments, the authors estimated that aldose reductase metabolizes 85% of glyoxal and 40% of MG at low dicarbonyl levels while the importance of GLO1 increases in the presence of high levels.

### 6.3. Aldehyde Dehydrogenase

The aldehyde dehydrogenase ALDH1A1 metabolizes MG by oxidation to pyruvate. However, the relative importance of this reaction in vivo is uncertain due to the presence of highly active GLO1 and AKR1B1 [120]. The importance for 3-DG metabolism is also in doubt since the major 3-DG metabolite in urine was identified to be 3-deoxyfructose, which is the product of aldose reductase [121]. Nevertheless, the 3-DG metabolite by ALDH1A1, 2-keto-3-deoxygluconic acid (Figure 4D), is increased in plasma and erythrocytes of diabetic patients [122]. In addition to erythrocytes, high activity levels of ALDH1A1 were found in lung, testis and liver [123]. ALDH1A1 is also capable of metabolizing glyoxal to glyoxylate but similar to 3-DG and MG the importance is uncertain [124]. Interestingly, ALDH1A1 also converts retinaldehyde to retinoic acid and knockdown of ALDH1A1 has been associated with browning of adipose tissue [125]. This effect was attributed to elevated levels of retinaldehyde. 

### 6.4. Fructosamine-3-kinase

One of the major pathways of AGE formation is via glucose-amine-adducts which in the case of lysine results in FL formation. FL can rearrange or fragment to form stable AGEs including CML, glucosepane and pentosidine. Phosphorylation of FL by fructosamine-3-kinase (FN3K) results in the destabilization of FL and the release of free lysine thus reversing the glycation process [88]. A mouse deficient of FN3K displayed significantly elevated intracellular FL residues supporting the relevance of the mechanism in vivo [126]. Interestingly, deficiency of FN3K was not associated with a phenotype under normoglycemic conditions and data in a diabetic model of these mice are lacking. One possible explanation for the lack of a phenotype is that the dicarbonyl 3-DG forms in the course of the deglycation process catalyzed by FN3K [88]. Consequently, activity of FN3K is likely to result in a shift from glucose derived AGEs to dicarbonyl derived AGEs. Hence the enzyme could even exacerbate the glycative stress due to the higher reactivity of dicarbonyls.

### 6.5. The Proteolytic System

While stable AGEs accumulate over time on proteins with a long half-life such as collagen of the skin, the AGE burden in organs that have higher protein turnover is shifted to a certain extent away from the modified protein to the proteolysis machinery. In this regard proteolysis is the only universal AGE detoxification mechanism. AGE modified proteins are more resistant to digestion by the proteasomal—as well as the lysosomal proteolytic system [127]. This does not necessarily result in accumulation of AGE-modified proteins as long as the proteolytic system is able to adapt the digestive capacity. In support for such an adaptation in diabetes, a decrease in glomerular basement membrane digestibility was associated with elevated proteolytic activity of the glomeruli in diabetic rats [128,129]. A correlation between AGE-collagen adducts, collagen digestibility and the lysosomal protease cathepsin L was present in diabetic and healthy rats as further evidence of an adaptive increase of the proteolytic capacity [65]. The role of cathepsin D and L was investigated in vitro and both proteases were increased upon stimulation with AGE-modified albumin which resulted in a protective effect [130]. Additional support that the proteolytic system has to deal with a greater burden of AGEs in diabetes than is suggested by AGE-protein adducts comes from the elevation of AGE free adducts. The latter stem at least in part from the proteolysis of endogenously modified proteins and are increased to a far greater extent than the protein adducts in type 1 diabetic patients (10-fold vs. 3-fold elevation of free MG-H1 vs. MG-H1-protein adduct) [131]. The general increase of proteolysis in type 1 and type 2 diabetic patients is likely to contribute to such an elevation of AGE free adducts [132,133].

## 7. Therapeutic Targeting of Dicarbonyls

Based on the formation pathway for AGEs, elevated blood glucose and dicarbonyl levels can be targeted for treatment. However, since intensive lowering of blood glucose levels in diabetes is associated with an increase in total mortality options in this regard are limited [134]. In addition, there is evidence that MG levels are increased independent of blood glucose levels so that targeting the blood glucose level may not inhibit the elevated formation of AGEs via the dicarbonyl pathway [69]. Different approaches have been tested to reduce the dicarbonyl load and AGE formation independent of the lowering of blood glucose levels.

### 7.1. Dicarbonyl Scavengers

Scavengers were the first drugs to be developed and tested in clinical trials to target the elevated dicarbonyl levels and results have been reviewed in [135]. Aminoguanidine was the prototype AGE inhibitor [136]. It reacts rapidly with MG, glyoxal and 3-DG via its guanidine group and is capable of lowering MG levels in rats [23,137]. The scavenger prevented the development of albuminuria in two studies in rats but no effect on albuminuria was seen in another study [138,139,140]. Positive effects of aminoguanidine were also seen on diabetic retinopathy [141]. While preclinical studies were promising overall, clinical trials of the compound were disappointing. A trial in type 1 diabetic patients (*A C*linical *T*rial *I*n *O*vert *N*ephropathy of Type *1* Diabetics, ACTION I) failed to reach the primary endpoint (time to doubling of serum creatinine) but aminoguanidine treated patients displayed lower levels of proteinuria [142]. A subsequent trial in type 2 diabetic patients (*A C*linical *T*rial *I*n *O*vert *N*ephropathy of Type *2* Diabetics, ACTION II) had to be terminated early due to an unfavorable perceived risk-to-benefit ratio [143]. Side effects included glomerulonephritis associated with high levels of autoantibodies in patients receiving high doses of the drug (2 × 300 mg per day) [142]. Additional issues with aminoguanidine are the rapid renal excretion as well as the induction of nitric oxide synthase [23,144,145].

The B6 vitamer pyridoxamine scavenges dicarbonyls in vitro and consequently has also been tested as inhibitor of dicarbonyl derived AGE formation [146]. In addition, pyridoxamine acts as a metal chelator and could prevent AGE formation via inhibition of the transition metal catalyzed autoxidation of glucose or FL [147,148]. Treatment with pyridoxamine normalized albuminuria and lowered CML and CEL levels in a type 1 and type 2 diabetic rat model [149,150]. The investigational drug also inhibited the development of retinopathy paralleled by lower CML levels in STZ-induced diabetes in rats [151]. A phase 2 study analyzing the treatment effect of pyridoxamine on diabetic nephropathy reported a reduction in serum creatinine in CML and CEL [152] but a second larger scale trial did not find any significant effect on serum creatinine levels [153]. A recent phase 3 study (Clinical Trials.gov: NCT02156843) was terminated preliminarily. One issue with pyridoxamine may be the scavenging kinetics. Two molecules of pyridoxamine are needed to inactivate one molecule of the dicarbonyl MG and the reactivity is relatively low [154,155]. Should the scavenging activity be sufficient for MG binding in vivo another hurdle in diabetic patients would be competition for binding with metformin. The latter compound is the current first-line therapy for type 2 diabetes and reacts covalently with dicarbonyls at similar rate as pyridoxamine [156,157].

Metformin is the current treatment of choice for type 2 diabetes due to its glucose lowering effect [134]. MG lowering effects of metformin have been reported in addition [158]. The mechanism behind the decrease in MG levels by metformin is uncertain. Metformin reacts with MG via its guanidine group but the reaction proceeds slowly [159]. Nevertheless, a MG-metformin adduct has been detected in urine of patients treated with the drug at concentrations up to 4.3 μM [156]. Lowering of MG levels by metformin is associated with an increase of GLO1 in peripheral blood mononuclear cells and a trend for increase in red blood cells [160]. Thus metformin may inhibit AGE formation directly through lowering of MG levels via two ways, namely MG scavenging and induction of GLO1 activity.

Alagebrium was designed as a compound to react with and break down existing AGEs rather than a scavenger of dicarbonyl compounds [161,162]. While alagebrium cleaves AGE cross-links in vitro there is evidence that the compound does not cleave cross-links in vivo [163]. Alternatively, alagebrium acts as a dicarbonyl scavenger and MG scavenging activity has been shown in rats and in vitro [164,165]. Positive effects were also seen on diabetic nephropathy in rats [166]. As opposed to pyridoxamine, clinical trials for alagebrium in diabetic and non-diabetic patients focused on macrovascular and cardiac related parameters such as blood pressure, left ventricular ejection fraction, left ventricular stiffness, endothelial function and arterial function [135]. While some clinical trials showed modest improvements, others were negative and currently no further trials are running.

### 7.2. Alternative Advanced Glycation End-Product Lowering Strategies

The induction of GLO1 is investigated as an alternative to scavengers to reduce dicarbonyl levels. GLO1 is regulated via binding of transcription factor Nrf2 to an antioxidant-response element (ARE) and as such activators of Nrf2 are capable of inducing GLO1 [167]. In a recent clinical trial of overweight and obese patients co-administration of the Nrf2 activators trans-resveratrol and hesperetin increased GLO1 activity in peripheral blood mononuclear cells [168]. This was associated with lower MG and fasting plasma glucose levels in a subgroup analysis of highly overweight patients (BMI > 27.5 kg/m^2^ and <30 kg/m^2^).

Another approach to tackle elevated AGE formation in diabetes is transition metal chelation. The homeostasis of transition metals is disturbed in diabetes which can contribute to dicarbonyl and AGE formation via autoxidative pathways [14,53,169,170]. Chelator treatment lowers AGEs, normalizes collagen metabolism and slows down cataract formation in diabetic rats [65,171]. Improved cardiac function and sciatic nerve motor conduction velocity in diabetic rats has been demonstrated upon chelator treatment [172,173]. In support for an ameliorative effect in humans a recent clinical trial reported that EDTA treatment reduced the risk of adverse cardiovascular outcomes particularly in a subgroup analysis of diabetic patients although AGE levels were not measured [174,175]. In another phase 2 clinical trial, administration of the copper chelator trientine over one year partially restored left ventricular hypertrophy in diabetic patients [176]. 

### 7.3. Future Perspectives for Therapeutic Advanced Glycation End-Product Targeting

A large number of animal and clinical trials have been carried out with the aim to target the AGE pathway in diabetes. While animal trials often showed positive effects clinical trials were mostly negative and none of the compounds has been approved for treatment. Reasons may in part lie in the dosing and pharmacokinetics of administered drugs. In pre-clinical trials drugs were often given at high doses with aminoguanidine and pyridoxamine administered at up to 1 g/kg/day in rats [139,148,150]. Doses of pyridoxamine and aminoguanidine in clinical trials were much lower at 100–500 mg/day (1.4–7.1 mg/kg/day at 70 kg body weight) and 100–600 mg/day (1.4–8.6 mg/kg/day) respectively, even though allometric scaling needs to be considered [135,177]. It is thus possible that doses were too low to result in a therapeutic effect. Moreover, as mentioned by Borg and Forbes, it is possible that targeting one pathway of AGE formation is not enough [135]. Treatment with dicarbonyl scavengers and GLO1 inducers does not prevent the transition metal catalyzed fragmentation of glucose and glucose lysine adducts and formation of associated AGEs. Similarly chelator treatment does not target the formation of reactive dicarbonyls from triosephosphates [24]. Moreover, while the pathway for glucosepane formation features a dicarbonyl intermediate it is unknown whether scavenging compounds are capable of reacting with these in vivo thus lowering the amount of cross-links [44]. 

An important question for future treatment strategies to reduce AGEs regards the reversibility of existing AGEs. AGE breakers were designed based on the idea that a compound must cleave previously formed AGEs to treat complications associated with elevated AGE levels [135]. This is not necessarily the case, as AGE modified proteins are cleared via protein turnover. Thus, if the rate of AGE formation is lowered, the amount of AGE-protein adducts is decreased over time. In the kidney and the heart which are affected by diabetic complications, collagen has an average age of 80 and 40 days respectively so that even AGE-collagen adducts could theoretically be decreased, if the rate of formation of new adducts is lowered [178]. This should at least be the case for AGEs with a short chemical half-life such as MG-H1. When targeting enzymes associated with deglycation or dicarbonyl metabolism, care needs to be taken to avoid unwanted side effects. Up regulation of FN3K to lower the amount of glucose-lysine adducts may lead to an increased dicarbonyl stress through 3-DG production [179]. Similarly, inhibition of aldose reductase to lower sorbitol levels may lead to elevated levels of MG, glyoxal as well as 3-DG [93]. In summary, new treatment strategies to reduce the dicarbonyl load and associated complications in diabetes include GLO1 inducer and improved scavenger molecules. A complementary strategy to reduce the dicarbonyl load via the autoxidative fragmentation of glucose and fructoselysine is available by transition metal chelation. 

## 8. Summary and Conclusions

The formation of dicarbonyls and resulting AGEs in diabetes are elevated and strongly associated with the development of complications. One reason for the therapeutic success of metformin may be that it interferes with dicarbonyl and AGE formation on several levels, namely by lowering blood glucose levels as well as the independent lowering of MG via scavenging and induction of glyoxalase 1. Consequently, future therapeutics for lowering dicarbonyls and AGE formation must be better at scavenging dicarbonyls or inducing GLO1 than metformin. Furthermore, alternative pathways for the reduction of AGE formation, such as transition metal chelation to reduce the autoxidative fragmentation of glucose could be targeted.

## Abbreviations

ACR	Albumin/creatinine ratio
AGE	Advanced glycation end-product
CEdA	*N*2-(1-carboxyethyl)-2′-deoxyadenosine
CEdG	*N*2-(1-carboxyethyl)-2′deoxyguanosine
CEL	*N*^ε^-(carboxyethyl)lysine
CML	*N*^ε^_-_(carboxymethyl)-lysine
FL	Fructoselysine
FN3K	Fructoseamine-3-kinase
GdG	3-(2′-Deoxyribosyl)-6,7-dihydro-6,7-dihydroxyimidazo-[2,3-*b*]purine-9(8)one
G-H1	Glyoxal-derived hydroimidazolone 1
Glucosepane	6-[2-{[(4*S*)-4-Ammonio-5-oxido-5-oxopentyl]amino}-6,7-dihydroxy-6,7,8,8a-tetrahydroimidazo[4,5-*b*]-azepin-4(5H)-yl]-l-norleucinate
GLO1	Glyoxalase 1
GLO2	Glyoxalase 2
GSH	Glutathione
HSA	Human serum albumin
MG	Methylglyoxal
MG-H1	Methylglyoxal-derived hydroimidazolone 1
MGdG	3-(2′-Deoxyribosyl)6,7-dihydro-6,7-dihydroxy-6/7-methylimidazo-[2,3-*b*]purine-9(8)one
Pentosidine	(2*S*)-2-Amino-6-[2-[[(4*S*)-4-amino-4-carboxybutyl]amino]imidazo[4,5-*b*]pyridin-4-yl]hexanoic acid
STZ	Streptozotocin
3-DG	3-Deoxyglucosone
3-DG-H1	3-Deoxyglucosone-derived hydroimidazolone 1
